# Supplementary material for: Design specifications and capabilities of a 3D simulation software for clinical education of physiotherapy students in the neurology department
Source: BMC Med Educ. 2024 Aug 29;24:944. doi: 10.1186/s12909-024-05547-3 (PMC11360853; doi:10.1186/s12909-024-05547-3)
Supplement: Supplementary file 1 — Supplementary Material 1 [file 12909_2024_5547_MOESM1_ESM.docx]

**Design Specifications and Capabilities of a 3D Simulation Software for Clinical Education of Physiotherapy Students in the Neurology Department**

**Sorayya Rezayi ^1^,** **Nastaran Ghotbi ^2, *^, Leila Shahmoradi ^3, *^, Zakiyeh Raisi Ardali ^4,^ , Haniyeh Choobsaz ^5^**

1. Assistant Professor of Medical Informatics, Department of Health Information Management, School of Paramedical Sciences, AJA University of Medical Sciences, Tehran, Iran.

**2, *. Corresponding author:** Professor of Physiotherapy, Department of Physiotherapy, School of Rehabilitation**,** Tehran University of Medical Sciences, Tehran, Iran. Email address: [nghotbi@tums.ac.ir](mailto:nghotbi@tums.ac.ir).

**3, *. Corresponding author:** Professor of Health Information Management, Health Information Management and Medical Informatics Department, School of Allied Medical Sciences, Tehran University of Medical Sciences, Tehran, Iran. Email address: Lshahmoradi@tums.ac.ir

1. M.Sc. of Medical Informatics, Health Information Management and Medical Informatics Department, School of Allied Medical Sciences, Tehran University of Medical Sciences, Tehran, Iran.
2. Ph.D. Candidate of Physiotherapy, Department of Physiotherapy, School of Rehabilitation**,** Tehran University of Medical Sciences, Tehran, Iran.

**Abstract**

**Introduction:** In this revolutionized era, thanks to cutting-edge technological breakthroughs like 3-dimensional (3D) computerized environments, physiotherapy trainers can improve their knowledge and confidence by using such training tools. Hence, there is room for developing these technologies for training medical students to expand their skills and expertise. This study aims to identify the design requirements and key functionalities of a 3D simulation software for the clinical education of physiotherapy students in neurology departments.

**Method:** First, by carefully reviewing neurological books, scientific articles, curriculum, and medical records, and consulting with experts, a scenario was compiled. In the next step, a researcher-developed questionnaire was designed. Then, experts' opinions were considered to confirm the validity and reliability of the questionnaire. The designed questionnaire was distributed among several neurological physiotherapists. Finally, the information elements, contents, and functional capabilities of the 3D software were determined by analyzing the data obtained from the questionnaire.

**Findings:** The main components for the design of physiotherapy educational software were identified based on the findings of the literature review, curriculum analysis, and medical record review. A survey of physiotherapy professors was conducted using a questionnaire created by the researcher in order to enhance the capabilities of simulation software and ascertain its primary functions. Following an analysis of the data from the distributed questionnaire, 37 essential features and contents have been proven to be more crucial than the rest for the creation of 3D simulation software. As a result, the essential and fundamental needs for the patient's training in reading their medical records and performing muscle strength assessments were recognized and extracted. Based on these findings, a researcher-developed scenario for the various real cases was then established. In the patient's medical record reading scenario, the student is required to read the patient's record in text format. Similarly, in the section on cranial nerves, pictures are utilised to reinforce the student's assessment skills in addition to textual content. Together with the audio and pop-up texts, the simulated 3D environment also offers training for the assessment of muscle strength.

**Conclusion:** As an educational tool, this software can enhance students' learning and assist in addressing the drawbacks of conventional teaching methods like lectures and hospital visits.

**Keywords:** Simulation, Clinical Education, Neurology, Physiotherapy

1. **Introduction**

Neurology is a branch of medicine that deals with the nervous system. Patients suffering from common neurological disorders such as stroke, Multiple Sclerosis (MS), and Parkinson’s disease [1, 2], face various activity and participation restrictions; physiotherapy is primarily involved in their management [3, 4]. So, it is necessary to train physiotherapists to effectively care for and treat people with neurological disorders. Indeed, the role of physiotherapists in the management and rehabilitation of neurological patients is very important [5, 6]. Physiotherapy students need different observational and analytical skills to enable them to evaluate the patient's neurological disorders in the clinical environment. A combination of conventional approaches and the use of computer-based technologies can play a major role in the education of physiotherapy students [7].

Previous studies on physiotherapy have reported on the use of haptic simulators, Virtual Reality (VR) simulators, computerized full-body manikins, paper vignettes, training devices specifically designed to teach a specific task, and simulated patients (either classmates, actors, or volunteers trained to play the role of a patient) [8] Computerized simulated learning environments are employed worldwide in professional education and health sciences, including physiotherapy, to teach specific attributes and skills. For physiotherapy students, using computer simulations can stimulate a variety of skills, including observing, measuring, forecasting, manipulating variables, and interpreting outcomes [9]. With the use of computers or smartphones, students can consciously cultivate metacognition, reflecting on their own learning, increasing their engagement and motivation in the classroom, and positioning themselves as powerful predictors [10]. These innovations are necessary to increase the capacity and maximize the effectiveness of education and ensure that all students enjoy standard learning experiences that cannot be guaranteed through traditional methods [11].

These new simulation technologies are now being used to replace time spent in actual clinical settings, help physiotherapy students learn new clinical procedures, close the knowledge gap between their prior and unique experiences, and advance their scientific understanding by applying knowledge in a quasi-realistic setting [12-14]. Standardization, fidelity (used in simulation to describe the degree of accuracy and imitation of the real sample), and repeatability in medical simulation have made these technologies usable in the field of clinical physiotherapy [15]. Furthermore, clinical high-tech simulation has many advantages as it increases knowledge, improves technical and communication skills, enhances student satisfaction, and promotes clinical decision-making [16, 17]. Additionally, it gives students a good foundation on which to build appropriate clinical experiences in a secure setting. It is important to remember that learning-oriented simulation tools cannot fully substitute actual patient learning experiences in a real-world clinical setting [18]. However, these technologies have reasonable potential to create scenarios for physiotherapy students in the field of interactive education with patients and their treatment, which provide organising clinical education. As evidence, physiotherapy students reported that the use of computerised simulation techniques enhanced their foundational understanding, clinical reasoning, practical skills, and interprofessional communication in a systematic review led by Rezayi et al. Students' learning levels improved, and their motivation and self-efficacy levels were noted to have increased after participating in computerized simulation learning. Additionally, these initiatives decreased the dependence on teachers  [10].

- 1. **Significance of this research**

Today, there is a sharp rise in the use of computerized simulation-based neurology training technologies. Students' medical knowledge and self-confidence in treating a variety of neurological conditions are enhanced by these technological training solutions for neurological diseases. Additionally, regardless of the underlying diagnosis, using simulation-based technologies to evaluate patients with neurological diseases advances our understanding of their activity restrictions [19, 20].

The main contribution of this research is to determine the essential features, system design specifications, and conceptual modeling of a 3D simulation software for physiotherapy students enrolling in neurology departments. This software can be used as an educational tool to improve students' learning and overcome the drawbacks of traditional teaching approaches like attending lectures and visiting hospitals.

1. **Materials and method**

This study considered three main steps to identify the design requirements and key capabilities of the software. The first step involved creating a training scenario based on a review of the literature and patient records for evaluating a patient suffering from a stroke. In the second step, the key contents and main requirements have been determined by conducting a questionnaire-oriented survey. In the third step, the conceptual model of the simulation software was designed by Unified Modeling Language (UML). A thorough description of the methodology is given in the subsections below.

- 1. **Compiling the scenario**
     1. **Reviewing records of real cases and literature**

Initially, to formulate the scenario of the process of the patient’s medical record reading and examining the muscle strength of the patient's limbs, the reference books and the contents of the internship module in the field of nervous system diseases (lesson code 47) in the undergraduate physiotherapy curriculum of Iran were reviewed and investigated. Then, to review the texts related to the design of similar computer software, the scientific articles published until March 2022 in databases such as Medline (through PubMed), Scopus, and Google Scholar were examined and reviewed. The keywords applied to retrieve scientific articles and books included a combination of keywords and medical subject titles (mesh) related to "physiotherapy," "training," "education," “students," "virtual reality," "augmented reality," "computer simulation," and "simulation training." Comprehensive details following a systematic literature review are published in our previous paper [21].

Accordingly, the medical records of some cases and real patients in the neurology department of Imam Khomeini Hospital Complex were investigated to create a scenario related to how to read records and extract information from records, including patient history, medical examinations, and diagnosis, which is the initial stage of physiotherapy evaluation. Thus, the research team crafted a scenario for evaluating a stroke patient.

For the physiotherapy examination, which includes muscle strength examination, sensory examination, balance, and gait examination, only the muscle strength examination was selected for this study. The reason for selecting strength examinations was to limit the project's size, and these examinations are considered the first level of evaluation in the physiotherapy treatment plan. As aforementioned, for preparing the contents of the scenario in this section, reference books related to muscle examination and functioning were investigated. Then, the position of the muscle strength examination was defined and adapted according to the condition of the patient hospitalized in the department (including the inability to be in a prone position, the inability to sit or stand, etc.) by a physiotherapy university professor with several years of clinical teaching experience in the department (inpatient setting). Thus, the scenario included two parts: (1) the patient’s record reading and (2) the examination of a stroke patient's muscle strength (upper and lower limbs).

- 1. **Determining design contents and specifications**

The second step involved determining the key contents and specifications for developing the 3D software. For this purpose, a researcher-developed questionnaire with 45 questions was created, developed, and validated based on the results of the first step (i.e., reviewing the texts and educational curriculum). The initial questionnaire consisted of three parts, including definitions of how to examine cranial nerves, medical record reading training, and physiotherapy examination training. Some meetings were conducted to come to a consensus with the eleven physiotherapist doctors who made up the expert panel to validate the questionnaire. Specifically, to ascertain the content validity of the questionnaire, two sets of questions (items) were answered. In the items related to the Content Validity Ratio (CVR), a three-option Likert scale was used to determine whether each item is "necessary," "useful but not necessary," or "not necessary." According to the number of experts who evaluated the items, the minimum CVR value had to be based on the Lawshe table. The Content Validity Index (CVI) was also used to measure the validity of the questionnaire. This index was presented by Waltz and Basel. To calculate the CVI, experts were asked to rate the relevance of each item based on a four-option Likert-like scale (not relevant, in need of major revision, relevant but in need of revision, relevant). In this way, the number of experts who chose options 3 and 4 was divided by the total number of experts, and if the resulting value was smaller than 0.7, the item had to be rejected; if it was between 0.7 and 0.79, the item had to be revised; and if it was larger than 0.79, the item had to be retained. Furthermore, the reliability of the questionnaire was confirmed via Cronbach's alpha of 96%.

After completing the above steps and summarizing the opinions of the research team, the items of the questionnaire were corrected, clarified, and finalized. At this point, a survey was sent to the eleven physiotherapist professors employed by Tehran universities to get their feedback. For this purpose, the final questionnaire with the title "Questionnaire for determining the content of simulation software for clinical education of physiotherapy students" was distributed among the professors. Due to the limitations of the study population, sampling was not done, and the entire population was considered for the study. A total of 18 questionnaires were sent to five professors in person (face-to-face) and 13 professors via email, from which 11 questionnaires were completed and returned. Notably, seven professors did not answer the questionnaires and did not submit their feedbacks.

- 1. **Conceptual modeling**

The data obtained from the completed questionnaires was analysed using SPSS statistical software version 26. From this analysis, the structural content of the 3D software was extracted. A conceptual model was designed based on the analysis. Furthermore, the conceptual model of the software and UML diagrams were designed using Visual Paradigm for UML 10.0. The outline of the implemented methodology is depicted in Figure 1.


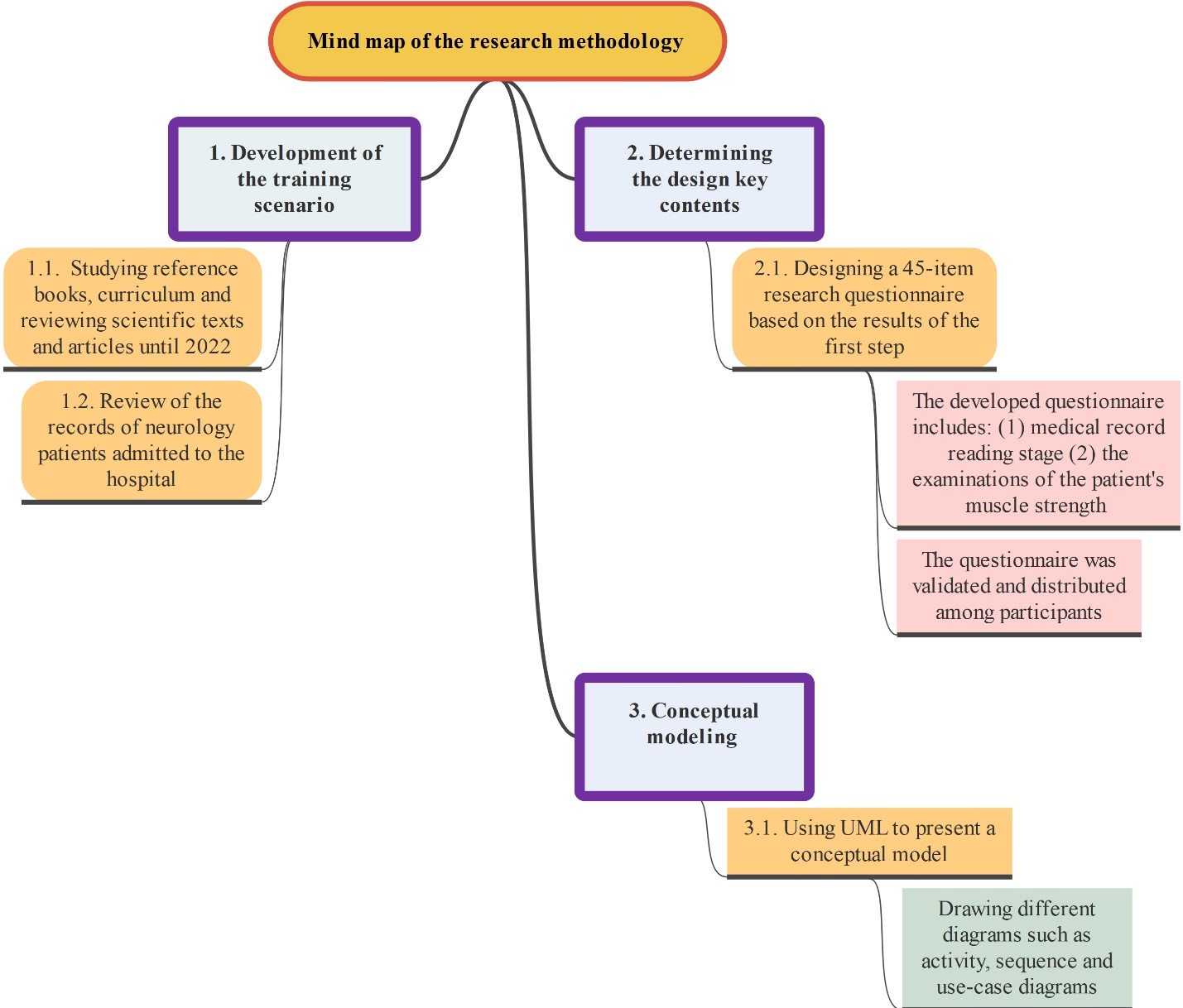


**Figure 1** Outline of the implemented methodology

- 1. **Ethical aspects**

All methods were carried out in accordance with relevant guidelines and regulations. The methodology for this study was approved by the Ethics committee of Tehran University of Medical Sciences (Ethical code IR.TUMS.SPH.REC.1400.038). All participants (or their legal guardians) were provided verbal informed consent for all stages of study and the Ethics committee of Tehran University of Medical Sciences approved this procedure.

1. **Results**

A total of 254 articles and books were found in the initial search. After removing duplicates, 233 articles remained. The authors reviewed the titles, abstracts, and keywords of selected articles, and after removing the papers that did not meet the requirements, 91 articles and books were identified for further review. After viewing the full text of these articles and books, focusing on the physiotherapy computerized simulation tools in the field of clinical education, and applying the entry criteria, 26 articles and some book chapters were finally reviewed [21]. Based on the investigations, the important and basic parameters of record reading training and muscle strength examination were identified and extracted, and then, for a real case, the researcher-developed scenario on how to carry out the work was prepared. The scenario for the record reading section is such that the user must read the patient record in text format, and for the cranial nerve section, in addition to the text, images are used to remind them how to perform the cranial nerve examination. The section on muscle strength examinations is also presented in the simulated 3D environment, along with the audio and pop-up texts.

The demographic characteristics of the participants in terms of gender, age, university degree, and training experience in the field of neurological diseases (outpatient or inpatient) are described in this work. Table 1 shows demographic information about the physiotherapy professors participating in the survey.

**Table 1** Demographic information of physiotherapy professors participating in the survey

| Variable | | Frequency | Frequency percentage (%) |
| --- | --- | --- | --- |
| Gender | Male | 7 | 63.63 |
|  | Female | 4 | 36.36 |
|  | Total | 11 | 100 |
| Training experience | Under 20 years | 4 | 36.36 |
|  | Between 20 and 30 years | 4 | 36.36 |
|  | Over 30 years | 3 | 27.27 |
|  | Total | 11 | 100 |
| University degree | All 11 participants had Ph.D. degrees in physiotherapy major. | | |

As shown in Table 1, the male participation rate (63.63%) was higher than that of women (36.36%). Participants with educational experiences below 20 years (36.36) and between 20 and 30 years (36.36) had a higher frequency than those with educational experiences above 30 years (27.27). Furthermore, all 11 physiotherapists had Ph.D. degrees and experience in teaching in the field of nervous system disorders. Figure 2 displays the number of professors from various universities.

**Figure 2** Frequency of physiotherapy professors recruited from different universities

The results of the analysis are presented in Table 2 in the following section. According to the data obtained from the questionnaires, the highlighted items were removed and not included; however, other items were identified as key contents and design capabilities of the software.

**Table 2** The mean scores, CVR and CVI for information elements and capacities of the software in the questionnaire

| ***#*** | ***Educational contents of medical record reading section*** | ***Mean of scores*** | ***CVR*** | ***CVI*** | ***#*** | ***Training on how to examine the cranial nerves (continued)*** | ***Mean of scores*** | ***CVR*** | ***CVI*** |
| --- | --- | --- | --- | --- | --- | --- | --- | --- | --- |
| 1 | The possibility of displaying the stage of receiving the record from the nursing station | - | 0.45 | 1 | 4 | 11^th^ pair of cranial nerves using image | 4.99 | 1 | 1 |
| 2 | The possibility of showing the student’s entrance to the patient's bedside | - | 0.09 | 0.90 | 5 | 12^th^ pair of cranial nerves using image | - | 0.45 | 0.90 |
| 3 | The possibility of displaying the record number | - | 0.27 | 0.90 | ***#*** | ***Physiotherapy examinations topics*** | ***Mean of scores*** | ***CVR*** | ***CVI*** |
| 4 | The possibility of displaying the patient's room and bed number | - | 0.45 | 0.90 | 1 | The possibility of displaying the physiotherapy request form | 3.89 | 0.81 | 1 |
| 5 | The possibility of displaying the name of the treating physician | - | 0.27 | 1 | 2 | The possibility to choosing the examination side by the user | 4.62 | 0.81 | 1 |
| 6 | The possibility of displaying the individual profile of the patient | 4.1 | 0.63 | 1 | 3 | The possibility of choosing upper and lower limbs | 4.28 | 0.81 | 1 |
| 7 | The possibility of displaying the main complaint and reason for referral | 4.02 | 0.81 | 1 | 4 | The possibility of displaying assessment goals and what is to be done | 4.18 | 0.81 | 1 |
| 8 | The possibility of displaying the current disease | 4.90 | 1 | 1 | 5 | The possibility of choosing the standard positioning of the patient | 4.23 | 0.81 | 1 |
| 9 | The possibility of displaying vital signs | 5 | 1 | 1 | 6 | The possibility of displaying an alarm to remind the patient's balance | 4.12 | 0.55 | 0.90 |
| 10 | The possibility of displaying the patient’s history | 4.88 | 0.81 | 1 | 7 | The possibility of displaying an alarm for the sensitivity of the correct placement of the physiotherapist's hand during the assessment | 4.19 | 0.55 | 0.90 |
| 11 | The possibility of displaying the mental state | 5 | 1 | 1 | 8 | The possibility of displaying the necessary explanations of the physiotherapist for the patient during the assessment | 4.01 | 0.63 | 0.90 |
| 12 | The possibility of displaying the examinations of cranial nerves | 5 | 1 | 1 | 9 | The possibility of showing the patient's inability to perform some movements | 5 | 1 | 1 |
| 13 | The possibility of displaying the status of reflexes | 5 | 1 | 1 | 10 | The possibility of entering the muscle strength grade by the user | 5 | 1 | 1 |
| 14 | The possibility of displaying motor system examinations | 5 | 1 | 1 | 11 | The possibility of displaying an alarm to inform the user about the wrong entry of the muscle strength grade | 3.79 | 0.81 | 0.90 |
| 15 | The possibility of displaying sensory system examinations | 5 | 1 | 1 | 12 | Teaching how to assess the muscle strength of proximal* segment of upper limb during shoulder abduction and shoulder adduction | 3.99 | 0.63 | 0.90 |
| 16 | The possibility of displaying cerebellar examinations | 5 | 1 | 1 | 13 | Training how to assess the muscle strength of middle* segment of the upper limb during elbow flexion and elbow extension | 3.93 | 0.63 | 0.90 |
| 17 | The possibility of displaying gait pattern | 5 | 1 | 1 | 14 | Training how to assess the muscle strength of distal* segment of upper limb during wrist extension and wrist flexion | 4.12 | 0.63 | 0.90 |
| 18 | The possibility of displaying a disease diagnosis | 4.93 | 1 | 1 | 15 | Training how to assess the muscle strength of proximal* segment of lower limbs during hip flexion and hip extension | 4.18 | 0.63 | 0.90 |
| ***#*** | ***Training how to examine the cranial nerves*** | ***Mean of scores*** | ***CVR*** | ***CVI*** | 16 | Training how to assess the muscle strength of the middle* segment of the lower limb during knee flexion and knee extension | 3.89 | 0.63 | 0.90 |
| 1 | The first pair of cranial nerves using image | - | 0.27 | 1 | 17 | Training how to assess the muscle strength of the distal* segment of the lower limb during dorsiflexion and plantarflexion | 3.98 | 0.63 | 0.90 |
| 2 | Second pair of cranial nerves using image | 4.12 | 0.63 | 1 | ***#*** | ***Other software features*** | ***Mean of scores*** | ***CVR*** | ***CVI*** |
| 3 | The third, fourth, and sixth pairs of cranial nerves using image | 4.55 | 0.63 | 1 | 1 | Providing a guide for using the system | 5 | 1 | 1 |
| 4 | The seventh pair of cranial nerves using the image | 5 | 1 | 1 | 2 | Providing the support contact information | 4.18 | 0.63 | 1 |
| 5 | The ninth and tenth pairs of cranial nerves using image | - | 0.45 | 1 | 3 | Providing information on the developers and the software provider team | 4.28 | 0.81 | 1 |

* Each limb was considered to have three segments: proximal, middle, and distal.

In this regard, the upper limb segments included shoulder, elbow, and wrist joints, respectively, and the lower limb segments included hip, knee, and ankle joints, respectively.

* The cells highlighted in orange are the excluded items according to the participants' opinions.

* The cells highlighted in blue show the main topics of the questionnaire.

Based on the statistical analysis, the orange-highlighted items were excluded from being used in the design of simulation software, and the experts rejected these items. Next, to produce and provide any software product with reliable, cost-effective, and efficient quality on real machines, it was necessary to use the principles of software engineering correctly and appropriately. The UML-integrated modeling language is an effective tool for software engineering and analysis. UML is a modeling language used to analyze and design object-oriented systems. UML diagrams include use case, activity, and sequence. Figures 3, 4, and 5 of the modeling diagrams are shown below:


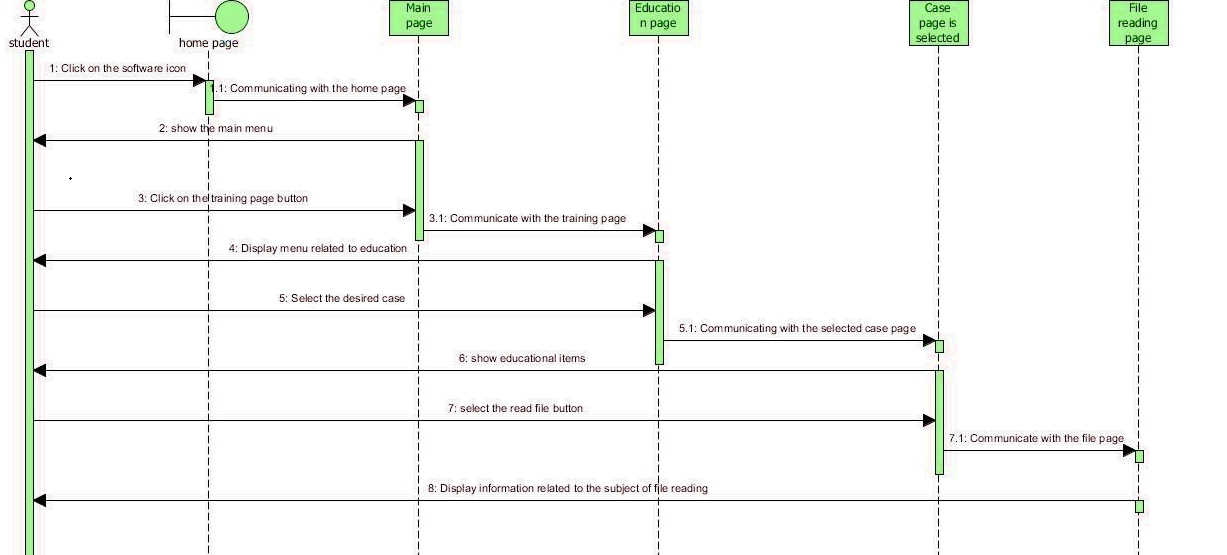


**Figure 3** Sequence diagram of the record reading process


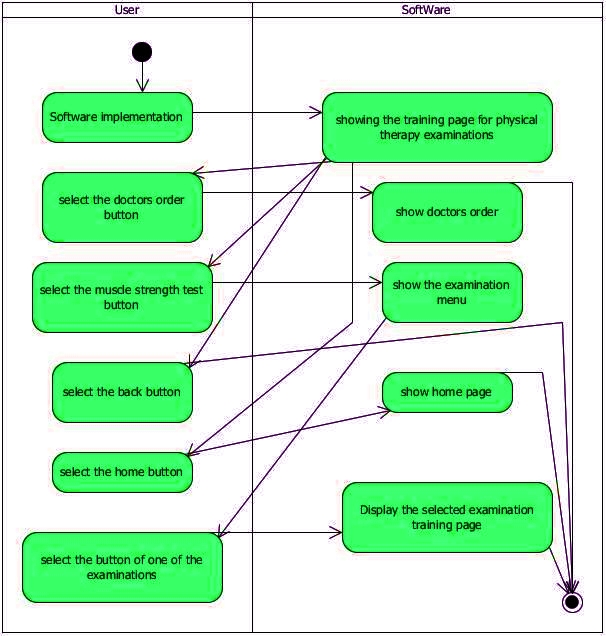


**Figure 4** The diagram of the physiotherapy examination activity


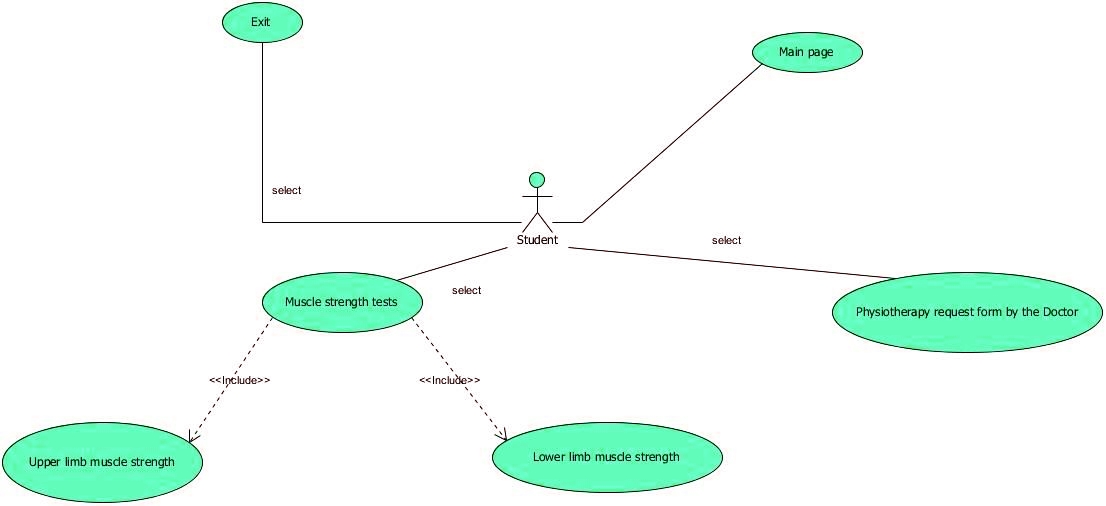


**Figure 5** The diagram of the physiotherapy examination application

1. **Discussion**

In this study, a scenario was compiled for a patient with the diagnosis of stroke. In the record-reading section of the 3D software, the user can read the patient's medical record in text format. In the second section, the muscle strength examinations and requirements are defined in the form of animation, text, and audio. Then, the content validity of the designated scenario was assessed by a questionnaire that contained the required items for various capabilities of the software in each section. Eleven university physiotherapy professors with Ph.D. degrees and an average educational experience of 20.81 years completed the questionnaire.

Based on CVR, the approved items for the first section included displaying information about reflex, motor, and sensory examinations, as well as gait patterns and the disease diagnosis. However, concerning the possibility of displaying cranial nerves, the experts did not approve of the necessity for displaying the information about the examination of the 1^st^,9^th^,10^th^, and 12^th^ cranial nerves. The necessity for displaying all items in the second section was approved by the experts.

One of the most important phases for designing simulation and educational software is to compile scenarios in detail [22, 23]. Consistent with our research, Ulrich F. and his colleagues (2021) conducted a study that emphasised the actual teaching environment in order to generate various scenarios. Consequently, the pertinent educational materials were developed in partnership with experts in physiotherapy education. The scenarios encompassed educational topics such as: (a) the significance of the supine position; (b) commonly used abbreviations; (c) proper placement of equipment; (d) accurate configuration and preparation of the medical bed; (e) correct positioning of the neck and knee rest; (f) techniques for achieving a locked knee in the supine position; and (g) strategies for managing patient restlessness [24]. In line with our study, significant scenarios were identified prior to the finalisation of the software's design and content determination [25].

Almost the same as our research, a framework was presented that aims to introduce a novel and flexible method for teaching in a simulation-based training environment in the medical sciences. The presented virtual operative assistant was validated for a complex neurosurgical procedure  [26]. In another study [27], similar to ours, there have been some training scenarios that are related to anatomy education. Users can engage in simulations where they can teleport to locations inside a virtual human body, resize and view objects from any angle, and sketch in a 3D environment to test their comprehension. Correspondingly, a framework for generating scenarios based on data was proposed for the purpose of game-based training. The findings indicated that taking into account the temporal aspect of events is crucial for evaluating scenarios, and the suggested framework effectively generates scenarios for game-based training [28]. Sequencing processes and events in scenarios enables the meticulous development of application software and ensures the design is based on sound principles. In our project, we systematically and scenario-orientedly compiled measures related to medical record reading and physiotherapy examinations to be used in the software development stage.

Skills other than record reading and examining the patient's muscle strength will be taught using the scenario that has been compiled. A specific scenario for an inpatient setting was defined for the current study so that students could gain experience managing a stroke patient during their first visit. In addition, not only can they apply their theoretical knowledge—which was intended for outpatient settings—but they can also examine six distinct segments in the upper and lower limbs in the proper positions according to the patient's circumstances. Training items such as the possibility of displaying the current disease, vital signs, the state of mind, the state of the cranial nerves, the status of reflexes, motor system examinations, sensory system examinations, cerebellar examinations, the walking condition, and all the other training items that are necessary to evaluate the patient's muscle strength are included in the developed scenario and the presented model.

Similar to our study, a visual platform was created to imitate gamified training situations using rapid prototyping and VR software design patterns. Scripting scenarios is the essential approach for creating and representing designs and models. A visual scripting module has the ability to create training applications using a node-based scripting system. The VR editor allows users/developers to customise and create new VR training scenarios directly within the virtual environment  [29].

We used a conceptual model for the 3D training software and UML diagrams for its analysis and design. UML consists of a series of graphic diagrams, and in this study, use-case, activity, and sequence diagrams were depicted. As can be seen in the diagrams of the physiotherapy simulation software, the student has been identified as the main factor in this software  [30]. Among the operations that are considered in these diagrams, we can point to entering the neurology department, medical record reading, muscle strength examinations of upper and lower limbs, and exiting the software  [31]. Since the use case depicted diagram could not fully show the components of activities, the activity diagrams were also used to model the workflow between the components of physiotherapy clinical education simulation software; for operations such as entering pages (training homepages, medical record reading, physiotherapy examinations, and exiting the software), activity diagrams have been depicted.

Due to the sequence diagrams' importance, a sequence diagram has been drawn for the physiotherapy clinical education simulation software, which included entering the pages of the training, record reading, physiotherapy examinations, and exiting the software.

- 1. **Strengths and limitations**

To the best of our knowledge, the current study is the first survey of experts to determine the information components and functional capabilities of the 3D software, which broadens the applicability of our findings. The unwillingness of some professionals who were referred to universities to cooperate is one of the study's most important shortcomings. The absence of relevant, useful literature was another significant barrier, yet our work inventiveness was incredibly high as a result of this dearth.

- 1. **Implications for practice**

In very recent years, traditional teaching approaches and learning methods have been supplanted by simulation-based learning tools. The need for computerized simulations is becoming more and more widespread worldwide, so modern countries are greatly affected by this problem. As a result, we suggest that developing nations provide an appropriate setting for this research. Though the cost, time, location, and methods of implementation vary widely based on the specifics of the application, the majority of them are costly and need adequate space. It is advised that governments budget for this kind of training and associated costs.

1. **Conclusion**

This software facilitates initial clinical training on neurophysiotherapy by offering an interactive and virtual environment. These discussions can involve reading medical records, obtaining pertinent information from patient records (such as history taking and other medical examinations), and performing physical therapy examinations, such as assessing a patient's muscle strength when they are admitted to the neurology department. This software, as an educational tool, enhances students' learning and can assist in addressing the drawbacks of conventional teaching methods like lectures and hospital visits. All it does is open up another avenue for more engaged learning. We just want to provide more training and learning opportunities so that students are better equipped for the clinical setting. Lectures and clinical placements will still take place for students. Future research investigations are necessary to create and develop this 3D software as well as to ascertain the effects of integrating 3D software into conventional training activities on skill performance, competency, retention, and patient safety.

**Declaration**

**Ethics approval and consent to participate:** All methods were carried out in accordance with relevant guidelines and regulations. The methodology for this study was approved by the Ethics committee of Tehran University of Medical Sciences (Ethical code IR.TUMS.SPH.REC.1400.038). All participants (or their legal guardians) were provided verbal informed consent for all stages of study and the Ethics committee of Tehran University of Medical Sciences approved this procedure.

**Consent for publication:** Not applicable.

**Availability of data and materials:** All data generated or analyzed during this study are included in this published article.

**Competing interests:** The authors declare no conflict of interest regarding the publication of this article.

**Funding:** In this paper, we didn't have any financial sponsors.

**Authors’ contributions:** Sorayya Rezayi, Nastaran Ghotbi, Leila Shahmoradi, Zakiyeh Raisi Ardali, Haniyeh Choobsaz: Conceptualization, Methodology, Software. Sorayya Rezayi, Nastaran Ghotbi, Leila Shahmoradi: Data curation, Writing- Original draft preparation. Sorayya Rezayi, Nastaran Ghotbi, Leila Shahmoradi, Zakiyeh Raisi Ardali, Haniyeh Choobsaz: Visualization, Investigation. Nastaran Ghotbi, Leila Shahmoradi: Supervision.

**Acknowledgement:** This paper is formed part of a thesis submitted by the fourth author (Z.RA) for an MSc degree at Tehran University of Medical Sciences.

***References***

1. Singh G, Rao D, Kumar A. Molecular Basis of Neurological Disorders. The Molecular Immunology of Neurological Diseases: Elsevier; 2021. p. 1-13.

2. Goetz CG. Textbook of clinical neurology: Elsevier Health Sciences; 2007.

3. Micieli G, Cavallini A, Santalucia P, Gensini G. Simulation in neurology. Neurological Sciences. 2015;36(10):1967-71.

4. Organization WH. Neurological disorders: public health challenges: World Health Organization; 2006.

5. Gustafsson L, Hutchinson L, Theodoros D, Williams K, Copley A, Fagan A, et al. Healthcare students’ experiences of an interprofessional, student-led neuro-rehabilitation community-based clinic. Journal of Interprofessional Care. 2016;30(2):259-61.

6. Davies A, Ramsay J, Lindfield H, Couperthwaite J. A blended approach to learning: added value and lessons learnt from students’ use of computer‐based materials for neurological analysis. British Journal of Educational Technology. 2005;36(5):839-49.

7. Blackstock FC, Watson KM, Morris NR, Jones A, Wright A, McMeeken JM, et al. Simulation can contribute a part of cardiorespiratory physiotherapy clinical education: two randomized trials. Simulation in Healthcare. 2013;8(1):32-42.

8. Chernikova O, Heitzmann N, Stadler M, Holzberger D, Seidel T, Fischer F. Simulation-based learning in higher education: a meta-analysis. Review of Educational Research. 2020;90(4):499-541.

9. Nsabayezu E, Iyamuremye A, Mukiza J, Mbonyiryivuze A, Gakuba E, Niyonzima FN, et al. Impact of computer-based simulations on students’ learning of organic chemistry in the selected secondary schools of Gicumbi District in Rwanda. Education and Information Technologies. 2023;28(3):3537-55.

10. Rezayi S, Shahmoradi L, Ghotbi N, Choobsaz H, Yousefi MH, Pourazadi S, et al. Computerized Simulation Education on Physiotherapy Students’ Skills and Knowledge: A Systematic Review. BioMed Research International. 2022;2022:4552974.

11. James S, Ma Z, Arrojo DR, Davison AJ. Rlbench: The robot learning benchmark & learning environment. IEEE Robotics and Automation Letters. 2020;5(2):3019-26.

12. Bøje RB, Bland A, Prescott S, Hannula L, Smith CM, Stirling K. The utilisation of a standardised educational framework to develop and deliver impactful programmes of simulation-based learning. Journal of Applied Learning & Teaching. 2024;6(1):74-84.

13. Bourne E, Bell A. Student perceptions of transferable skills learned during a simulated placement. Innovations in Education and Teaching International. 2024:1-15.

14. Shahmoradi L, Almasi S, Ghotbi N, Gholamzadeh M. Learning promotion of physiotherapy in neurological diseases: Design and application of a virtual reality-based game. Journal of Education and Health Promotion. 2020;9(1):234.

15. Watts PI, McDermott DS, Alinier G, Charnetski M, Ludlow J, Horsley E, et al. Healthcare simulation standards of best practiceTM simulation design. Clinical Simulation in Nursing. 2021;58:14-21.

16. Ayed A, Malak MZ, Alamer RM, Batran A, Salameh B, Fashafsheh I. Effect of high fidelity simulation on clinical decision-making among nursing students. Interactive Learning Environments. 2023;31(4):2185-93.

17. Robinson BK, Dearmon V. Evidence-based nursing education: Effective use of instructional design and simulated learning environments to enhance knowledge transfer in undergraduate nursing students. Journal of professional nursing. 2013;29(4):203-9.

18. So HY, Chen PP, Wong GKC, Chan TTN. Simulation in medical education. Journal of the Royal College of Physicians of Edinburgh. 2019;49(1):52-7.

19. Blümcke I, Arzimanoglou A, Beniczky S, Wiebe S, Force ET. Roadmap for a competency‐based educational curriculum in epileptology: report of the Epilepsy Education Task Force of the International League Against Epilepsy. Epileptic Disorders. 2019;21(2):129-40.

20. Oliveira L, Figueiredo E. Simulation training methods in neurological surgery. Asian Journal of Neurosurgery. 2019;14(02):364-70.

21. Rezayi S, Shahmoradi L, Ghotbi N, Choobsaz H, Yousefi MH, Pourazadi S, et al. Computerized Simulation Education on Physiotherapy Students’ Skills and Knowledge: A Systematic Review. BioMed Research International. 2022;2022.

22. Siu CY, O'Brien W, Touchie M, Armstrong M, Laouadi A, Gaur A, et al. Evaluating thermal resilience of building designs using building performance simulation–A review of existing practices. Building and Environment. 2023;234:110124.

23. Zaher AA, Hussain GA, Altabbakh H. An Active Learning Approach for Applying STEAMeD-Based Education in Engineering Programs. International Journal of Engineering Pedagogy. 2023;13(3).

24. Ulrich F, Helms NH, Frandsen UP, Rafn AV. Learning effectiveness of 360 video: experiences from a controlled experiment in healthcare education. Interactive Learning Environments. 2021;29(1):98-111.

25. Brown AR, Voltz BD. Elements of effective e-learning design. International Review of Research in Open and Distributed Learning. 2005;6(1):1-10.

26. Mirchi N, Bissonnette V, Yilmaz R, Ledwos N, Winkler-Schwartz A, Del Maestro RF. The Virtual Operative Assistant: An explainable artificial intelligence tool for simulation-based training in surgery and medicine. PloS one. 2020;15(2):e0229596.

27. Kolla S, Elgawly M, Gaughan JP, Goldman E. Medical student perception of a virtual reality training module for anatomy education. Medical Science Educator. 2020;30:1201-10.

28. Luo L, Yin H, Cai W, Zhong J, Lees M. Design and Evaluation of a Data-Driven Scenario Generation Framework for Game-Based Training. IEEE Transactions on Computational Intelligence and AI in Games. 2017;9(3):213-26.

29. Zikas P, Papagiannakis G, Lydatakis N, Kateros S, Ntoa S, Adami I, et al. Immersive visual scripting based on VR software design patterns for experiential training. The Visual Computer. 2020;36(10):1965-77.

30. Munn Z, Aromataris E, Tufanaru C, Stern C, Porritt K, Farrow J, et al. The development of software to support multiple systematic review types: the Joanna Briggs Institute System for the Unified Management, Assessment and Review of Information (JBI SUMARI). JBI Evidence Implementation. 2019;17(1):36-43.

31. Bianchi M, Marzi G, Guerini M. Agile, Stage-Gate and their combination: Exploring how they relate to performance in software development. Journal of Business Research. 2020;110:538-53.
